# In Patients Experiencing Pain and Inflammation, the Use of Non-Steroidal Anti-Inflammatory Drugs Is More Likely a Marker of the Observed Cardiovascular Risk Rather than Its Cause: A Viewpoint

**DOI:** 10.3390/pharmacy14060139

**Published:** 2026-09-21

**Authors:** Gerhard Zingler, Thomas Herdegen

**Affiliations:** 1 Medical Department, MSD Sharp & Dohme GmbH, 81673 Munich, Germany; gerhard.zingler@freenet.de; 2 Institute of Experimental and Clinical Pharmacology, University Hospital Schleswig-Holstein Campus Kiel, Hospitalstrasse 4, 24105 Kiel, Germany

**Keywords:** cardiovascular risk, NSAIDs, osteoarthritis, rheumatoid arthritis, systemic inflammation, meta-analysis

## Abstract

Non-steroidal anti-inflammatory drugs (NSAIDs) have been among the most frequently prescribed medications worldwide for several decades. Ongoing vigilance regarding their potential adverse effects on the gastrointestinal tract and kidneys remains critical. Over the past twenty years, accumulating evidence has indicated an increased cardiovascular (CV) risk associated with NSAID use, posing a significant challenge for both patients and healthcare providers. This issue has prompted the US Food and Drug Administration (FDA) to issue explicit warnings concerning NSAID use. Recent data and comparative analyses of existing publications provide a basis for critically re-evaluating the association between NSAID use and potential CV risk, particularly when NSAIDs are administered for approved indications such as the treatment of pain and inflammation in patients with arthritis. The debate regarding the CV safety of NSAIDs centers on establishing causality. It is necessary to determine whether NSAIDs directly induce CV risks by initiating new pathological processes or act as modulators that influence the severity of pre-existing susceptibilities. The evidence presented in this review supports the view that NSAIDs do not create new CV risks but reduce CV incidents in patients with inflammatory pathologies such as arthritis. Conversely, NSAIDs have been observed to slightly increase CV risk when used at inappropriate high doses or in patients without inflammatory pain, such as those with Alzheimer’s disease or other vulnerable populations. This perspective shifts the focus from causality to context-based risk management. The distinction between risk generation and risk modulation is proposed as a framework for the rational use of NSAIDs. The potential for NSAIDs to exacerbate pre-existing CV risk is dependent on the context of morbidity, and is low when used for anti-inflammatory indications in populations with the highest benefit, i.e., those with inflammatory morbidities. A general condemnation of NSAIDs keeps relevant patient populations from the only anti-inflammatory analgetic pharmacotherapy so far available.

## 1. Introduction

NSAIDs rank among the most frequently prescribed and self-administered medications globally, providing relief from pain, inflammation, and fever in a wide range of acute and chronic conditions. In rheumatology, NSAIDs are commonly employed to manage persistent joint pain and swelling [1]. These agents are indicated for various forms of arthritis, including osteoarthritis (OA), rheumatoid arthritis (RA), and other joint disorders [2]. Additionally, NSAIDs are frequently purchased over the counter for ailments such as headaches, toothaches, menstrual pain, and other pain syndromes, including back pain [3].

Non-selective NSAIDs are commonly prescribed analgesics; their adverse effects on the gastrointestinal tract (GIT) and kidneys are well established. GTI complications frequently result from NSAID-induced inhibition of cyclooxygenase-1 (COX-1), an enzyme that ensures gastric mucosal integrity. The loss of this protective mechanism increases the risk of irritation, ulceration, and gastrointestinal bleeding. Renal complications arise due to prostaglandin inhibition, which diminishes renal blood flow and may precipitate acute kidney injury, particularly in older adults or individuals with pre-existing renal impairment.

For over two decades, concerns regarding CV safety have also been prominent. Specifically, discussions about the increased risk of CV events have generated significant uncertainty among both patients and healthcare providers, prompting the US Food and Drug Administration (FDA) to issue a formal warning about NSAID use in 2015 [4].

In this boxed warning, the FDA states that this class of drugs:

- May cause an increased risk of heart attack and stroke.
- This risk may occur within the first few weeks of NSAID therapy.
- Appears to pose a greater risk at higher doses.
- The risk may increase with the duration of NSAID use.
- Increases the risk in patients with and without heart disease, with a higher risk in those with existing heart disease or cardiovascular risk factors.
- Is associated with a higher risk of death in patients who have already suffered a heart attack if they are treated with NSAIDs after the event.
- Increases the risk of heart failure when taking NSAIDs.

The FDA warnings are informed by both observational studies and randomized controlled trials, which were reviewed during a joint meeting of the Arthritis Advisory Committee and the Drug Safety and Risk Management Advisory Committee on 10–11 February 2014 [4].

Recent reviews suggest an association between NSAID use and CV events; however, definitive evidence remains insufficient. Current data indicate that NSAIDs do not independently cause CV risk but may exacerbate existing risk factors. This perspective shifts the focus from establishing causality to prioritizing risk mitigation strategies. Consequently, revisions to clinical guidelines may be warranted [5,6].

This review critically evaluates the current evidence on the CV safety of NSAIDs, including pharmacological mechanisms, clinical trial outcomes, and patient-specific risk factors. The studies ensured that gender-specific factors did not account for differences in CV risk of NSAIDs. However, in women—particularly after the menopause—differences in the distribution, metabolism and clearance of NSAIDs lead to greater functional COX-2 inhibition, which may in itself influence cardiovascular risk compared with men [7]. By differentiating between risk creation and risk modulation, a framework is proposed for rational NSAID use that treats CV risk as a management issue rather than an inevitable outcome of therapy.

## 2. Mechanism of Action of NSAIDs

NSAIDs exert their pharmacological effects primarily by inhibiting cyclooxygenase (COX) enzymes, which catalyse the conversion of arachidonic acid to prostaglandins and thromboxane. Two principal isoforms have been identified: COX-1, which is constitutively expressed in most tissues and is essential for maintaining gastrointestinal mucosal integrity, renal perfusion, and platelet function; and COX-2, which is inducible and predominantly mediates inflammatory responses. NSAIDs reduce the synthesis of pro-inflammatory prostaglandins, such as PGE_2_ and PGI_2_, by inhibiting these enzymes, thereby exerting analgesic and anti-inflammatory effects. Traditional NSAIDs (tNSAIDs) are non-selective, inhibiting both COX-1 and COX-2, which frequently leads to gastrointestinal side effects, particularly at higher doses or with prolonged administration. In contrast, selective COX-2 inhibitors have been developed to provide effective anti-inflammatory action while reducing the gastrointestinal adverse effects associated with tNSAIDs, including inhibition of platelet aggregation. Selective COX-2 inhibitors (coxibs) preferentially inhibit the synthesis of pro-inflammatory prostaglandins, including prostacyclin PGI_2_ and prostaglandin PGE_2_, while preserving thromboxane TXA_2_ production by platelets [1,8,9].

The Vioxx^®^ Gastrointestinal Outcomes Research (VIGOR) trial was the first study to demonstrate an increased risk of myocardial infarction (MI) associated with a COX-2-specific inhibitor. The trial compared the gastrointestinal (GI) safety of rofecoxib and naproxen in patients with RA [10]. Although rofecoxib was associated with a lower risk of GI events and similar CV mortality rates compared to naproxen, the naproxen group experienced significantly fewer CV events, primarily myocardial infarctions (MIs), than the rofecoxib group [10].

Analysis of the VIGOR results led to the hypothesis that maintaining haemostatic balance between prostacyclin (PGI_2_) and thromboxane (TXA_2_) is essential. These counter-regulatory mediators are believed to exert opposing effects, suggesting that equilibrium between the antithrombotic and vasodilatory prostacyclin PGI_2_ (COX-2) and the proaggregatory and vasoconstrictive thromboxane TXA_2_ (COX-1) is necessary [1,8].

Cardiovascular adverse effects associated with NSAID therapy are primarily attributed to an imbalance between PGI_2_ and TXA_2_. This mechanism indicates that such adverse effects are not limited to COX-2-specific inhibition, as an elevated risk of CV disease is also observed with tNSAIDs such as diclofenac and ibuprofen. In the case of tNSAIDs, the imbalance is thought to result from their inhibition of both COX-1 and COX-2 to varying degrees, whereas coxibs are believed to preferentially suppress endothelial PGI_2_ synthesis without altering platelet TXA_2_ production [1,8,9].

The use of COX-2-specific inhibitors and tNSAIDs has generated significant debate due to concerns regarding CV safety. Multiple studies, systematic reviews, and large-scale meta-analyses of randomized controlled trials (RCTs) and observational studies have consistently demonstrated an association between these medications and an increased risk of CV events. Comparisons frequently assess NSAIDs against each other or against a placebo [11,12]. Nevertheless, some analyses have not identified a significant CV risk associated with standard doses of NSAIDs [13,14].

## 3. Results of NSAID Studies Showing an Association with Cardiovascular Risk

At the FDA meeting, a variety of studies were reviewed, thus providing a range of perspectives on the potential CV risks associated with the use of NSAIDs. The majority of these studies were large, population-based cohort and case–control analyses, which consistently demonstrated a moderate increase in the incidence of myocardial infarction (MI), stroke, and heart failure among NSAID users compared to non-users [4]. Notably, three recent meta-analyses (MAs), considered among the most comprehensive to date, synthesized data from numerous RCTs and observational studies to elucidate the comparative CV risks of individual NSAIDs [15,16,17].

**Figure 1:**
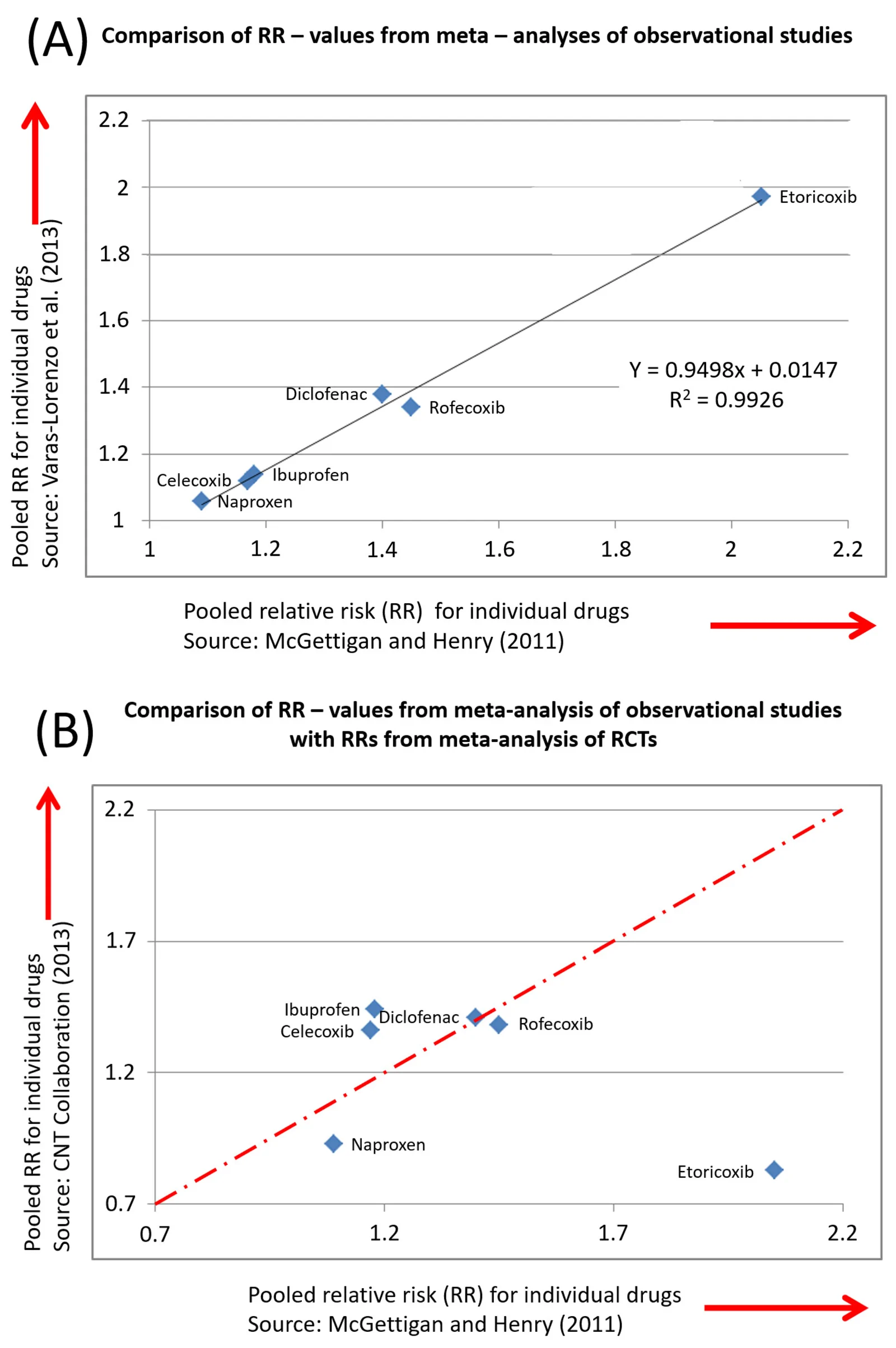
A comparison of the cardiovascular risks of different tNSAIDs and COX-2 selective inhibitors is presented. (**A**) depicts two distinct meta-analyses of observational studies [15,16], while (**B**) illustrates findings from observational studies with randomized clinical trials [15,17].

Figure 1A demonstrates that the findings of the two MAs of observational studies are highly consistent, which constitutes a significant observation. McGettigan et al. [15] assessed the overall CV risk associated with NSAIDs, including multiple endpoints, whereas Varas-Lorenzo et al. [16] focused specifically on the risk of acute myocardial infarction (AMI). The estimated risks for AMI in both studies were similar in magnitude and trend to those reported in previous MAs of observational studies [14,18]. In contrast, the third MA, which utilized data from RCTs, did not align with the findings of the observational study MAs (Figure 1B). Only the relative risk values for diclofenac and rofecoxib were close to the equivalence line.

Therefore, the reliability of these data regarding the CV safety of NSAIDs is uncertain. For instance, MAs of observational studies indicate an elevated CV risk for etoricoxib (RR 2.05, 95% CI 1.45–2.88) [15], whereas the risk reported in the MA of RCTs is substantially lower (RR 0.83, 95% CI 0.18–3.77) [17].

Bhala et al. [17] conducted an MA presented at an FDA meeting that included several sub-analyses. Although the overall population exhibited an increased risk of heart attacks, this risk was not significantly elevated among patients with arthritis. The observed increase in risk for the overall population was primarily attributable to studies investigating COX-2 inhibitors for cancer and Alzheimer’s disease prevention [17].

Previous research has demonstrated that the CV risk associated with NSAID use varies according to patient characteristics. An MA by Salpeter et al. [19] found that tNSAIDs did not significantly impact CV events (odds ratio [OR] 1.3; 95% confidence interval [CI] 0.8 to 2.1). Subgroup analyses revealed no increased CV risk in studies involving joint diseases (OR 0.6; 95% CI 0.2 to 1.7). In contrast, a consistently higher CV risk was observed in studies of patients with Alzheimer’s disease (OR 1.6; 95% CI 0.9 to 2.7) [19,20,21,22]. Scott et al. [23] reported comparable results, indicating no substantial association between NSAID use and myocardial infarction (MI) in case–control studies (odds ratio [OR] 1.17; 95% confidence interval [CI] 0.99–1.37) or in cohort studies of patients with arthritis (relative risk [RR] 1.03; 95% CI 1.00–1.07). Conversely, four RCTs evaluating NSAID use for the prevention of colon adenomas identified a significantly increased risk of MI (RR 2.68; 95% CI 1.43 to 5.01) [23].

The reliability of studies examining NSAIDs for the prevention of colon adenoma and Alzheimer’s disease remains inconclusive. Both the aspirin study by Baron et al. [24] and the ADAPT study [25] involving naproxen reported increased CV risk, despite these agents generally being considered cardioprotective [17,26].

An MA by De Vecchis et al. [27] compared the vascular risks of the COX-2 inhibitors celecoxib and etoricoxib. The analysis found an increased risk of MI in patients treated with celecoxib compared to placebo. In contrast, studies assessing etoricoxib for musculoskeletal disorders did not report an increased risk of serious vascular events relative to placebo. Notably, all studies on Alzheimer’s disease and colorectal adenomas used celecoxib exclusively [27].

NSAIDs are approved for the treatment of pain and inflammation, but not for the prevention or inhibition of colorectal adenomas or the development of Alzheimer’s disease [21,28]. In the context of clinical relevance, the population exhibiting approved inflammatory indications does not demonstrate an enhancement in CV risk.

## 4. Meta-Analysis of Observational Studies: No Convincing Results

Two MAs of observational studies [15,16] report that both COX-2-specific inhibitors and tNSAIDs are associated with a 30 to 40 percent increased risk of CV events. Naproxen is associated with a relatively low risk of AMI (relative risk [RR] 1.06, 95% confidence interval [CI] 0.94–1.20). In contrast, celecoxib (RR 1.12, 95% CI 1.00–1.24) and ibuprofen (RR 1.14, 95% CI 0.98–1.31) are linked to a modestly elevated risk, whereas rofecoxib (RR 1.34, 95% CI 1.22–1.48) and diclofenac (RR 1.38, 95% CI 1.26–1.5) are associated with a higher risk [16].

Interpretation of these results requires caution, as they are based on comparisons between NSAID users and non-users in population-based cohort and case–control studies. The findings may be affected by confounding factors such as indication bias, comorbidities, and over-the-counter use. According to speakers at the FDA meeting, the observational studies discussed were not randomized, and a causal relationship between NSAID use and outcomes has not been definitively established. Furthermore, many relevant studies lack detailed clinical information and data on prognostic factors, including left ventricular ejection fraction (LVEF), body mass index (BMI), blood pressure, smoking status, and lipid levels. In observational research, the specific indication for treatment is often unknown or subject to indication bias, and unmeasured confounding factors may further influence the results [4].

Wolfe et al. [29] conducted the first comprehensive analysis of bias in observational studies. In their research, arthritis patients treated with a new COX-2 inhibitor had a longer history of side effects, experienced more severe pain and functional limitations, and demonstrated greater use of inpatient and outpatient services compared to those who continued with tNSAIDs. The measured variables among these patients increased by approximately 25%, primarily due to indication and channeling bias. This bias led to the selection of patients with more severe clinical profiles for COX-2 inhibitor treatment [29].

A cohort study by Garcia Rodriguez et al. [30] highlights another limitation of observational studies, particularly within nested case–control analyses. The study reported that NSAID use was associated with increased CV risk in patients presenting with unexplained chest pain (odds ratio [OR] 6.33, 1.37–29.30), but not in patients with OA (OR 1.03, 0.83–1.29), who constituted the majority of the study population [30]. This outcome, attributed to protopathic bias, is especially pertinent for NSAIDs, which are commonly administered for acute pain [16].

Shaya et al. [31] assessed the CV risk associated with COX-2-specific inhibitors compared to tNSAIDs in a high-risk population through an observational cohort study. Their methodology differed from other observational studies by employing a logistic model to match the control cohort based on disease risk score, thereby ensuring a robust and comparable dataset. The propensity-adjusted odds ratio indicated that COX-2 inhibitor use did not significantly influence CV risk in this patient group (odds ratio, 1.09; 95% confidence interval, 0.90–1.33). These findings suggest that COX-2 inhibitors do not increase CV risk relative to non-naproxen NSAIDs in high-risk populations [30].

Van Staa et al. [32] conducted a large retrospective cohort study to evaluate the risk of AMI associated with tNSAID use, while accounting for exposure patterns. The study demonstrated a statistically significant increase in the risk of MI among current diclofenac users (RR = 1.21) compared with the control group. In contrast, no elevated risk was observed for current users of ibuprofen (RR = 1.04) or naproxen (RR = 1.03). Stratification by the number of previous prescriptions revealed no differences in MI risk patterns among the three NSAIDs [32].

A Danish study [33] presented at the FDA meeting [4] identified an association between NSAID use and increased CV risk in individuals classified as “healthy” compared to non-users. The reported hazard ratios (95% confidence intervals) for death or MI were: ibuprofen, 1.01 (0.96–1.07); diclofenac, 1.63 (1.52–1.76); naproxen, 0.97 (0.83–1.12); rofecoxib, 2.13 (1.89–2.41); and celecoxib, 2.01 (1.78–2.27). Participants were considered healthy if they had no history of hospitalisation and were not receiving specific pharmacotherapy [33]. However, the study’s methodology has been criticised by other researchers [34,35,36], and these findings should be interpreted with caution due to limited data on CV events.

Generally, individuals classified as “healthy” do not require regular or prolonged use of NSAIDs. An exception occurs in sports disciplines, where painkillers may be misused to prevent the onset of pain, as recently described [37].

Warnings about CV risks associated with NSAID use in healthy adults are frequently based on correlational studies rather than mechanistic or interventional evidence, making them susceptible to indication bias and other confounding factors. The case of patients with inflammatory joint diseases exemplifies this challenge. Nielen et al. [38] identified elevated C-reactive protein (CRP) levels in healthy blood donors who later developed RA, with these markers most commonly detected within two years before symptom onset and showing significant increases in individuals with preclinical RA [38]. Additional studies have reported that musculoskeletal symptoms, infections, and comorbidities are more common in patients with inflammatory arthritis than in control subjects during the years preceding diagnosis. As a result, these patients more often sought care from general practitioners for pain-related conditions [39]. Maradit-Kremers et al. [40] compared a cohort of RA patients with matched controls and found that individuals with RA were more frequently hospitalized for heart failure immediately before diagnosis and had a higher incidence of sudden death. These findings suggest that some individuals who met the diagnostic criteria for RA may have died before a formal diagnosis was established [40].

Rahme and Nedjar [41] examined the relationship between NSAID use and the risk of hospitalization for AMI. Hazard ratios (HR) were consistently higher among patients who also received aspirin. For instance, the adjusted HR for rofecoxib users was 1.14 (1.00–1.31), whereas the HR for those using both rofecoxib and aspirin was 1.28 (1.08–1.51) [41]. These findings, along with results from other studies, underscore the challenges of interpreting observational research due to potential confounding factors. Additionally, patients treated with aspirin continue to exhibit elevated rates of cardiac events, raising questions about the extent of aspirin’s protective effects [41,42].

This pattern may also apply to NSAID therapy, since individuals experiencing pain and inflammation frequently have an elevated baseline cardiovascular risk prior to NSAID administration [13,14,43,44,45,46,47]. The increased CV risk seen in patients requiring NSAID treatment may not be due solely to NSAID use. In such cases, the need for treatment with NSAIDs is more indicative of an existing CV risk than of the drugs themselves causing these side effects.

## 5. Studies of NSAIDs in Approved Indications

MAs of RCTs and observational studies have produced conflicting results concerning the risks associated with NSAIDs. While observational data are frequently regarded as reflective of real-world patient experiences, significant discrepancies remain between these findings and those from randomized trials. This review evaluates MAs that investigate the CV safety of NSAIDs in patients with OA and RA, which are the primary approved indications for these medications.

The study by van Walsem et al. [48] is significant because it was the first to integrate multiple endpoints, including efficacy (pain relief, physical functioning, patient global assessment (PGA)), tolerability (treatment discontinuation), and the most frequently examined risks associated with NSAIDs (gastrointestinal and cardiovascular). Additionally, the analysis compared various benefits and risks within a relatively homogeneous patient population, specifically patients with arthritis.

Fabule and Adebajo examined the CV outcomes of active substances currently available and commonly used in Europe, specifically tNSAIDs and COX-2 inhibitors, administered at approved dosages in patients with RA and OA [49].

Gunter et al. [50] conducted an MA evaluating eight NSAIDs, including three non-selective NSAIDs (nsNSAIDs) and five COX-2 inhibitors (coxibs), both individually and in direct comparison, to determine whether CV risk is associated with COX-2 selectivity. The analysis considered datasets both including and excluding rofecoxib to assess potential bias from this drug. The study included 24 RCTs and 2 prospective cohort studies, comparing NSAIDs either head-to-head or against placebo. The majority of included studies focused on patients with OA and RA [50].

The MAs found no significant differences in CV risk among the NSAIDs assessed. This result may reflect the homogeneity of the arthritis patient population. Gunter et al. [50] also reported that none of the NSAIDs demonstrated a statistically significant effect on CV disease-related mortality. Excluding rofecoxib from the analysis revealed no differences among COX-2 inhibitors, suggesting that rofecoxib may have influenced the overall findings [50]. Most data on rofecoxib were derived from the VIGOR study, which did not include aspirin. The MA provided no evidence that selective COX-2 inhibition modifies CV risk [50].

Only two RCTs, the MEDAL study [51] and the PRECISION study [52], have specifically examined the CV risk associated with NSAID use. These investigations were prompted by concerns, following the VIGOR study, that certain COX-2 inhibitors might increase CV side effects. The MEDAL study compared the CV safety of the COX-2-specific inhibitor etoricoxib with diclofenac, while the PRECISION study compared celecoxib with ibuprofen and naproxen. Both studies demonstrated that the CV safety profile of COX-2 inhibitors is comparable to that of tNSAIDs [51,52]. Neither the MEDAL programme, which enrolled 34,701 patients, nor the PRECISION study, with 24,081 participants, included a placebo group due to ethical considerations. In both studies, the incidence of CV events was higher in the intention-to-treat population than in the per-protocol or on-treatment populations [51,52]. Consequently, when used to treat pain and inflammation (their primary and intended indications), NSAIDs may reduce rather than increase the risk of CV events in patients with OA and RA. This hypothesis has also been put forward elsewhere [53,54]. Furthermore, the PRECISION study confirms this hypothesis in arthritis patients with an increased risk of CV disease [55]. Despite this focus, the observed rate of CV events remained low, at approximately 1% per year [52]. This observation suggests that NSAID use may contribute to the prevention of CV events.

## 6. Studies on NSAIDs in Patients with Systemic Inflammation

Comparative analyses of heart attack risk among patients with various forms of arthritis indicate that individuals with RA have a significantly higher risk (RR: 1.69, 95% CI: 1.50 to 1.90) than those with OA (RR: 1.31, 95% CI: 1.01 to 1.71). The risk observed in patients with ankylosing spondylitis (AS) (RR: 1.24, 95% CI: 0.93–1.65) is similar to that in OA patients [56]. Additional studies demonstrate that individuals with inflammatory arthritis generally face an increased risk of developing heart disease [57,58]. Furthermore, RA patients exhibit a CV risk comparable to individuals who are 10 years older, a level of risk similar to that observed in patients with diabetes mellitus [59].

RA patients have a higher CV risk than those with OA. However, when both RA and OA patients receive NSAIDs, RA patients experience a lower CV risk than those with OA [53,54]. Studies involving RA and AS patients further indicate that lower NSAID intake, as opposed to higher intake, is associated with increased CV mortality [53,54]. The incidence of CV events in inflammatory diseases increases after discontinuation of NSAID therapy [60]. The increased risk of CV morbidity and mortality in RA patients, relative to OA patients, can be attributed to several factors, including traditional CV risk factors, disease activity, systemic inflammation, and the type and intensity of pain [53,54,61].

Inflammation is widely recognized as a significant pathogenetic factor in CV diseases, contributing to the development of atherosclerosis, heart failure, and aortic stenosis. Additionally, the role of inflammation in pain among patients with RA is well established. Clinical findings and laboratory data demonstrate that inflammatory reactions precede pain and that the severity of inflammation is positively correlated with pain intensity [62].

Roubille et al. [63] conducted the first MA to assess the impact of anti-inflammatory drugs on CV events in inflammatory diseases, including RA, psoriasis, and psoriatic arthritis. The analysis found that treatment with TNF inhibitors or methotrexate in RA was associated with a 30% and 28% reduction in CV event risk, respectively. In contrast, the use of NSAIDs or corticosteroids was associated with an 18% and 47% increase in the risk of all CV events, respectively [63]. Methotrexate and TNF inhibitors suppress inflammatory mediators to a greater extent than NSAIDs (RR 1.18; 95% CI 1.01 to 1.38; *p* = 0.04). Only long-term NSAID use has been linked to cardioprotective effects in patients with arthritis and systemic inflammation [54,63].

Several large-scale cohort studies of patients with inflammatory arthritis have demonstrated that long-term NSAID use (up to 10 years) for pain and inflammation does not increase the risk of CV events. This finding contrasts with the FDA’s 2015 warning, which noted increased CV risk from long-term NSAID use [4,54].

Individuals with inflammatory arthritis have an elevated risk of CV disease, yet may derive therapeutic benefits from NSAIDs [53,54]. Several studies report cardioprotective effects associated with prolonged NSAID use [54]. Research on celecoxib dosage indicates a lower risk of coronary heart disease among individuals taking more than 300 mg per day compared to non-users, with the highest doses correlating with the lowest risk [64,65]. Hung et al. [66] examined the risk of coronary heart disease (CHD) in RA patients treated with celecoxib and etoricoxib, finding a lower incidence of CHD in etoricoxib users than in those taking celecoxib. Despite this, celecoxib is frequently identified as the safest COX-2-specific inhibitor in other observational studies [16,54,63].

Braun et al. [53] propose that NSAIDs may elevate the risk of CV events in individuals using them long-term for pain management, according to observational studies. Conversely, NSAIDs may reduce CV risk in patients with chronic inflammatory diseases. Lindhardsen et al. [67] further suggest that NSAIDs could serve as a marker, rather than a direct cause, of increased CV risk in active rheumatoid arthritis.

## 7. Is It Time to Reconsider the FDA’s Warnings from 2015?

Although observational studies have associated NSAID use with increased CV risk [18], these results may be influenced by missing data, methodological limitations, and confounding by indication. The inherent limitations of observational research raise questions regarding the factors that influence the prescription of specific drugs to higher-risk patients as opposed to others [14,15,16,42,54].

The meta-analysis by the Coxib and Traditional NSAID Trialists’ Collaboration (CNT) evaluated cardiovascular and gastrointestinal risks associated with specific NSAID therapies in diverse patient populations [17]. The results of this network meta-analysis were based on indirect comparative analyses, a method that has been criticized for its vulnerability to error [68]. Despite these methodological concerns, the publication is widely cited and served as a key reference during the 2014 FDA meeting [4]. Table 1 is adapted from Bhala et al. [17]. The original table contained transposed entries for history of atherosclerosis (Yes/No) and aspirin use (Yes/No); these entries have been corrected. The data in Table 1 indicate that older patients (aged 60 years and above), those with a history of atherosclerosis, individuals with an elevated five-year risk of major vascular events (MVE), and patients using aspirin have a lower CV risk when treated with COX-2 specific inhibitors (coxibs) compared to tNSAIDs. Furthermore, indication-specific data from the CNT meta-analysis show that COX-2 inhibitor studies targeting Alzheimer’s disease and colorectal adenomas are associated with a higher CV risk than those focused on arthritis [17,19,20,21,22,25,53].

Furthermore, both the MEDAL and PRECISION studies reported a higher rate of CV events in the intent-to-treat (ITT) population compared to the per-protocol population [51,52]. This finding indicates that CV risk may depend on the population analysed and supports the conclusion that NSAID therapy, when used as directed for approved indications such as arthritis, is not associated with increased CV risk [53,54].

Additionally, several studies investigating the association between NSAID use and CV events in patients with inflammatory arthritis have found no increase in CV risk in this population. Instead, NSAID use has been linked to a potential cardioprotective effect [53,54,69].

The FDA has indicated that CV risks associated with NSAID use may emerge early during treatment [4]. However, a large study on postoperative pain management found NSAIDs to be both safe and effective. The study’s authors recommend considering NSAIDs as a viable alternative to opioids [70]. Furthermore, evidence indicates that prolonged NSAID use following total hip replacement reduces the incidence of heterotopic ossification and is associated with a low risk of complications, supporting a favourable risk-benefit profile for prophylactic NSAID use in hip surgery [71].

A recent review article on anti-inflammatory drug therapy for patients with CV disease indicates that acute and recurrent pericarditis are primarily managed according to established protocols that utilize NSAIDs or aspirin in combination with colchicine [72]. Another recent publication demonstrated the long-term effectiveness of this therapeutic approach. Among patients hospitalized for acute pericarditis, post-discharge ibuprofen use was not linked to an increased risk of CV complications in either the short or long term, compared to non-use. These findings further support the safety of NSAIDs in current clinical practice [73].

A study of data from 42,394 female patients aged between 55 and 74 was conducted to evaluate the relationship between cancer incidence and ibuprofen usage. The results indicated that women who consumed at least 30 ibuprofen tablets per month exhibited a lower risk of endometrial cancer (HR: 0.75; 95% CI: 0.58–0.98). The reduction in risk was more pronounced (HR: 0.57; 95% CI: 0.37–0.87) among women with a history of CV disease [74].

A recent comprehensive network MA of RCTs examined various anti-inflammatory therapies for reducing CV events in patients with known CHD. The use of NSAIDs was associated with a significant reduction in major adverse cardiac events (MACE) in patients with acute coronary syndromes (OR: 0.30, 95% credible interval (CrI): 0.11–0.74) and stable CHD (OR: 0.64, 95% CrI: 0.43–0.96) [75].

A re-evaluation of NSAID use in patients with inflammatory arthritis is warranted for several reasons. The prevailing hypothesis suggests that COX-2-specific NSAIDs elevate CV risk by disrupting the balance between prostacyclin (COX-2) and thromboxane A2 (COX-1), resulting in a prothrombotic state. However, a recent safety update highlights that this theory is based on a study in which COX-2-specific NSAIDs increased urinary thromboxane metabolites and decreased prostacyclin metabolites. Importantly, paracetamol demonstrated similar effects in a previous study using the same methodology, yet it was not considered prothrombotic [76].

Furthermore, experimental evidence does not support the theory of an imbalance in prostaglandin homeostasis. Recent studies indicate that, under physiological conditions, COX-1 is primarily responsible for prostacyclin production in the CV system, rather than COX-2. Additionally, urinary prostacyclin measurements do not accurately reflect endogenous prostacyclin production in systemic circulation [77].

Additionally, research on prostacyclin as both a physiological and pathological mediator and a therapeutic agent has demonstrated its dual role as an inflammatory mediator and a homeostatic regulator in the CV system [78]. Prostacyclin (PGI_2_) exhibits paradoxical effects in inflammatory diseases: it provides protection against inflammation in atherosclerosis but may promote inflammatory responses in rheumatic diseases such as RA and OA [79]. This duality may account for the inconsistent findings reported in various studies and supports the concept of the ‘paradoxical role of prostacyclin.’ Numerous MAs have found no correlation between NSAID use and CV risk in patients with joint disease [17,19,53,54]. In contrast, increased CV risk has been observed in patients with Alzheimer’s disease, who do not experience pain or inflammation, following prolonged NSAID use [17,19,20,25]. These findings underscore the importance of adhering to approved indications for NSAIDs, specifically the management of pain and inflammation in patients with OA and RA.

A cohort study comparing the safety of three major classes of analgesics (tNSAIDs, coxibs, and opioids) in older adults with arthritis concluded that NSAIDs were safer than opioids, with coxibs demonstrating the lowest all-cause mortality [80].

## 8. Conclusions

A range of novel research findings demands a re-evaluation of the utilisation of NSAIDs. Current guidelines report that NSAID administration increases the risk of CV events, contributing to heart attacks, strokes, and related outcomes regardless of baseline risk. However, the association between NSAIDs and CV events observed in observational studies may be significantly affected by indication bias and other confounding variables prevalent among NSAID users, such as advanced age, inflammation, pain, or comorbidities. Although prolonged use of COX-2 inhibitors for delaying Alzheimer’s disease or preventing colorectal adenomas is associated with an increased risk of CV disease, appropriate NSAID use for OA and RA is linked to a reduced risk of this. Research indicates that individuals suffering from systemic inflammatory diseases such as AS and RA experience CV benefits from NSAID therapy.

Consequently, it is of greater pertinence to differentiate between the targeted population (inflammatory morbidities versus non-inflammatory diseases) than to generally limit the prescription of NSAIDs. It is imperative that healthcare professionals consider the potential benefits of reducing CV risk in patients diagnosed with inflammatory OA or RA when formulating treatment plans. The substitution of NSAIDs with glucocorticoids or opioids constitutes an inappropriate replacement, which has the potential to increase the risk of severe adverse effects. Conversely, the inappropriate cessation of NSAIDs can lead to an escalation in pain, inflammation, triggered damages and comorbidities.

These findings highlight the necessity for high-quality, contemporary studies and comprehensive MAs to inform future recommendations regarding NSAID use in patients with approved indications.

The uses of NSAIDs now extend beyond pain relief. They are regarded as versatile molecular tools that influence inflammatory pathways, stress signals, epigenetic programmes and viral dynamics. These functions are becoming increasingly important in cancer therapy and virological research. Further studies are needed to ensure their safety and, above all, to identify comorbidities that could increase the risk of side effects [81].

## Figures and Tables

**Table 1:** Comparisons of Coxibs vs. Non-Naproxen NSAIDs and Coxibs vs. Naproxen. Effect on Major Vascular Events by Baseline Characteristics.

	Rate Ratio (95%CI)			Rate Ratio (95%CI)		
Subgroup	Coxib vs. Placebo	Coxib vs. tNSAIDs	Adjusted Rate Ratio for tNSAIDs vs. Placebo	Coxib vs. Placebo	Coxib vs. Naproxen	Adjusted Rate Ratio for Naproxen vs. Placebo
**Indication for Treatment**						
Arthritis	1.25 (0.72, 2.16)	1.01 (0.88, 1.16)	**1.15 (0.55–2.44)**	1.25 (0.72, 2.16)	1.62 (1.22, 2.14)	**0.63 (0.28–1.41)**
Cancer	1.63 (1.21, 2.18)	NE	**NE**	1.63 (1.21, 2.18)	NE	**NE**
**Age, Years**						
<60	1.44 (0.87, 2.39)	1.33 (0.95, 1.86)	**1.08 (0.51–2.30)**	1.44 (0.87, 2.39)	1.99 (1.13, 3.51)	**0.72 (0.28–1.89)**
≥60	1.44 (1.15, 1.79	0.94 (0.81, 1.09)	**1.53 (1.09–2.16)**	1.44 (1.15, 1.79)	1.45 (1.08, 1.96)	**1.03 (0.65–1.63)**
**History of Atherosclerosis**						
Yes	1.34 (0.91, 1.99)	0.97 (0.74, 1.26)	**1.41 (0.77, 2.58)**	1.34 (0.91, 1.99)	1.39 (0.82, 2.35)	**1.04 (0.46–2.35)**
No	1.48 (1.17, 1.88)	1.01 (0.86, 1.18)	**1.45 (1.01–2.10)**	1.48 (1.17, 1.88)	1.65 (1.21, 2.24)	**0.91 (0.56–1.48)**
**Five Years MVE Risk**						
<5%	1,69 (1.17, 2.44)	1.10 (0.90, 1.34)	**1.50 (0.88–2.55)**	1.69 (1.17, 2.44)	1.57 (1.04, 2.37)	**1.10 (0.54–2.23)**
>10%	1.48 (1.06, 2.07)	0.81 (0.60, 1.10)	**1.83 (1.04–3.22)**	1.48 (1.06, 2.07)	1.19 (0.72, 1.98)	**1.27 (0.60–2.69)**
**Aspirin User**						
Yes	1.33 (0.78, 2.25)	0.96 (0.77, 1.19)	**1.38 (0.68–2.79)**	1.33 (0.78, 2.25)	1.38 (0.79, 2.38)	**1.04 (0.41–2.64)**
No	1.40 (1.07, 1.82)	1.03 (0.85, 1.26)	**1.36 (0.89–2.08)**	1.40 (1.07, 1.82)	1.76 (1.29, 2.40)	**0.81 (0.48–1.35)**

The results are taken from the publication of Bhala et al. [17]. NE = not estimated, MVE = major vascular events (non-fatal myocardial infarction, non-fatal stroke, or vascular death).

## Data Availability

No new data were created or analyzed in this study. Data sharing is not applicable to this article.
